# Potential interactions between the TBX4-FGF10 and SHH-FOXF1 signaling during human lung development revealed using ChIP-seq

**DOI:** 10.1186/s12931-021-01617-y

**Published:** 2021-01-21

**Authors:** Justyna A. Karolak, Tomasz Gambin, Przemyslaw Szafranski, Paweł Stankiewicz

**Affiliations:** 1 Department of Molecular & Human Genetics, Baylor College of Medicine, One Baylor Plaza, Rm ABBR-R809, Houston, TX 77030 USA; 2 Chair and Department of Genetics and Pharmaceutical Microbiology, Poznan University of Medical Sciences, 60-781 Poznan, Poland; 3 Institute of Computer Science, Warsaw University of Technology, 00-665 Warsaw, Poland

**Keywords:** Transcriptional regulation, Motif enrichment, Lung morphogenesis

## Abstract

**Background:**

The epithelial-mesenchymal signaling involving SHH-FOXF1, TBX4-FGF10, and TBX2 pathways is an essential transcriptional network operating during early lung organogenesis. However, precise regulatory interactions between different genes and proteins in this pathway are incompletely understood.

**Methods:**

To identify TBX2 and TBX4 genome-wide binding sites, we performed chromatin immunoprecipitation followed by next-generation sequencing (ChIP-seq) in human fetal lung fibroblasts IMR-90.

**Results:**

We identified 14,322 and 1,862 sites strongly-enriched for binding of TBX2 and TBX4, respectively, 43.95% and 18.79% of which are located in the gene promoter regions. Gene Ontology, pathway enrichment, and DNA binding motif analyses revealed a number of overrepresented cues and transcription factor binding motifs relevant for lung branching that can be transcriptionally regulated by TBX2 and/or TBX4. In addition, TBX2 and TBX4 binding sites were found enriched around and within *FOXF1* and its antisense long noncoding RNA *FENDRR,* indicating that the TBX4-FGF10 cascade may directly interact with the SHH-FOXF1 signaling.

**Conclusions:**

We highlight the complexity of transcriptional network driven by TBX2 and TBX4 and show that disruption of this crosstalk during morphogenesis can play a substantial role in etiology of lung developmental disorders.

## Background

Transcription factors (TFs) are essential proteins regulating gene activity through sequence-specific DNA binding. Coordinated cooperation between multiple TFs is critical for the specificity and maintenance of different cell types during development, including formation of the respiratory tree [1]. The initial stages of lung organogenesis rely mainly on epithelial-mesenchymal crosstalk controlled by diverse TF families [2] and include the Sonic hedgehog—Forkhead box protein F1 (SHH-FOXF1) and T-box transcription factor 4—Fibroblast growth factor 10 (TBX4-FGF10) signaling pathways [3].

FOXF1 (MIM# 601089), a member of the Forkhead Box family of TFs, is an important target of the SHH signaling [4]. The transcriptional activation of *Foxf1* in mice has been shown to be induced through the SHH-regulated GLI TFs [5]. In humans, transcription of *FOXF1* on 16q24.1 is strongly regulated by a distant lung-specific enhancer located ~ 270 kb upstream to *FOXF1* [6]. Heterozygous copy-number variant (CNV) deletions of *FOXF1* and/or their upstream regulatory region or point mutations in *FOXF1* have been reported in patients with a lethal lung developmental disorder (LLDD), alveolar capillary dysplasia with misalignment of pulmonary veins (ACDMPV; MIM# 265380) [7].* Foxf1* heterozygous knockout mice die at the early embryonic stage due to primarily lung developmental defects [5, 8]. Interestingly, homozygous loss of the *Foxf1* antisense long non-coding RNA (lncRNA) gene, FOXF1 adjacent non-coding developmental regulatory RNA (*FENDRR*, MIM# 614975) has also been demonstrated to be perinatal lethal due to multiple abnormalities of heart, lung, and gastrointestinal tract [9, 10].

Recently, the T-box transcription factor 2 (TBX2) and TBX4 TFs, and the ligand FGF10, known to be regulated by SHH epithelial-mesenchymal signaling during lung development [11, 12], have been associated with LLDDs other than ACDMPV. Heterozygous single nucleotide variants (SNVs) in *TBX4* (MIM# 601719) or CNV deletions involving *TBX4* and its neighboring *TBX2* (MIM# 600747) on 17q23.2 have been described in newborns with acinar dysplasia (AcDys), congenital alveolar dysplasia (CAD), or other primary pulmonary hypoplasias in newborns [13–15]. In support of this notion, homozygous loss of *Tbx2* or *Tbx4* in mice results in reduced lung branching [12, 16]. Heterozygous SNVs in *TBX4* and CNV deletions involving *TBX4* and *TBX2* have been reported also in pediatric and adult patients with pulmonary arterial hypertension (PAH) [17, 18], ischiocoxopodopatellar syndrome (MIM# 147891), and developmental delay, heart defects, and limb abnormalities [19]. The observed extrapulmonary anomalies likely result from disruption of the TBX2/TBX4 pathway as these genes are widely expressed, including heart and limbs [20, 21]. Recently, homozygous variants involving *TBX4* have been associated with posterior amelia with pelvic and pulmonary hypoplasia syndrome (MIM# 601360). Decreased levels of *Tbx4* and T-box transcription factor 5 (*Tbx5*) have been shown to suppress *Fgf10* expression in developing murine lung, suggesting that *Fgf10* is likely a downstream target of TBX4 [16]. Corroboratively, homozygous *Fgf10* knockout is neonatal lethal due to complete disruption of branching morphogenesis [22] and heterozygous SNVs or CNV deletions involving *FGF10* on 5p12 have been found in newborns with AcDys, CAD, or other pulmonary hypoplasias [15]. Interestingly, heterozygous variants in *FGF10* have been described previously in patients with milder phenotypes—aplasia of lacrimal and salivary glands (MIM# 180920) and lacrimo-auriculo-dento-digital syndrome (MIM# 149730).

Even with recognizing these members of the tissue-specific transcriptional network operating during early lung development, precise regulatory interactions between different TFs and their target genes in developing human lung are incompletely understood. In these studies, we examined a genome-wide distribution of the TBX2 and TBX4 binding sites in human fetal lung fibroblasts IMR-90 using chromatin immunoprecipitation followed by next-generation sequencing (ChIP-seq).

## Methods

### Cell culture

Human fetal lung fibroblasts IMR-90 (ATCC, Manassas, VA, USA) were cultured in Eagle's Minimal Essential Medium (ATCC) supplemented with 10% fetal bovine serum (ATCC) and 1% Penicillin–Streptomycin mixture (100 units/ml) at 37 °C in a humidified atmosphere containing 5% CO_2_. IMR-90 cell line derives from the lungs of a 16-week fetus and has been used widely in various studies aiming to identify the regulatory elements in the human genome, e.g. ENCODE and Roadmap. Since lung autopsy tissue does not offer best quality specimen and weeks 8–17 of gestation are crucial for lung branching, we elected this cell line was the most optimal system to study mechanistically TBX2 and TBX4 regulation during human lung development [12, 16].

### Chromatin immunoprecipitation and next-generation sequencing (ChIP-seq)

Chromatin immunoprecipitation was done using the EZ-Magna ChIP™ A/G kit (Merck-Millipore, Burlington, MA, USA). Briefly, intact cells were fixed using 1% formaldehyde (Sigma-Aldrich, St. Louis, MO, USA), followed by cell and nuclear lysis, according to the manufacturer’s instructions. Cross-linked DNA was then sheared to ~ 200–1000 bp fragments using Q125 Sonicator (Qsonica, Newtown, CT, USA) with the following pulse mode settings: 10 s with 50 s cooling, amplitude 30%, 8 cycles. DNA/protein complexes were immunoprecipitated overnight using antiTBX2 (D-3) X and antiTBX4 (F-12) X antibodies (Santa Cruz Biotechnology, Dallas, TX, USA). Mouse IgG antibody (Merck-Millipore) was used as a negative control. Protein-DNA immune complexes and input DNA (cross-linked and sonicated but not immunoprecipitated sample) were decross-linked, and deproteinized according to the manufacturer’s protocol. DNA was extracted using a spin filter column (Merck-Millipore). The sequencing and generation of short DNA reads were carried out at CloudHealth Genomics (Shanghai, China) using the HiSeqX platform (Illumina, San Diego, CA, USA).

### ChIP-seq data analyses

FASTQ files were processed using bcbio chip-seq pipeline that includes adapter trimming with atropos, mapping with bwa mem (v. 0.7.17), and peak calling with MACS2 (v. 2.2.6). Obtained peaks were further analyzed using ChipSeeker R package [23] and custom R scripts. Enriched peaks were annotated to the nearest ENSEMBL Release 100 (April 2020) gene and gene biotypes extracted using Biomart data mining tool [24].

### Comprehensive motif analyses

The MEME-ChIP within MEME Suite 5.1.1 [25] was used to determine novel long (up to 30 nt; MEME tool) and short (up to 8 nt; DREME tool) overrepresented DNA binding motifs in the TBX2 and TBX4 peaks, and analyze them for similarity to the known binding motifs (TOMTOM tool). The software was run for 100 nt sequences from a peak summit using default parameters. The AME tool was implemented to identify known motifs that show enrichment for particular location within TBX2 and TBX4 peak summits.

### Functional enrichment analyses

Functional annotation of the neighboring genes associated with the ChIP-seq peaks for TBX2 and TBX4 was determined by Gene Ontology (GO) enrichment analyses [26, 27] using PANTHER online tool [28]. The GO terms with a corrected p-value < 0.05 (FDR) were considered significantly enriched. The overrepresentation analysis of molecular pathways among genes surrounding TBX2 and TBX4 peaks was implemented using the ConsensusPathDB tool [29]. Only pathways with p-value ≤ 0.05 and sharing at least two genes with the ChIP-seq gene sets were analyzed.

### Intersection of TBX2 and TBX4 peaks with known super-enhancers for IMR-90

Bedtools ‘Intersect intervals’ tool in Galaxy (Version 2.29.0) platform [30] were used to compare the positions of enriched peaks identified for TBX2 and TBX4 with known super-enhancers intervals, previously detected in IMR-90 cells [31].

## Results

### TBX2 and TBX4 target genes in human fetal lung fibroblasts

To detect the binding sites for TBX2 and TBX4 in IMR-90 cells, chromatin was immunoprecipitated with their antibodies followed by sequencing of both samples along with DNA from the non-immunoprecipitated input. A total of 10,958,104, 8,191,590, and 2,958,900 aligned fragments were identified for TBX2, TBX4, and input DNA, respectively. After data normalization, 14,322 and 1862 strongly-enriched binding sites with a 150 bp peak size for TBX2 and TBX4 were detected (Additional file 1), including 991 overlapping intervals.

**Fig. 1:**
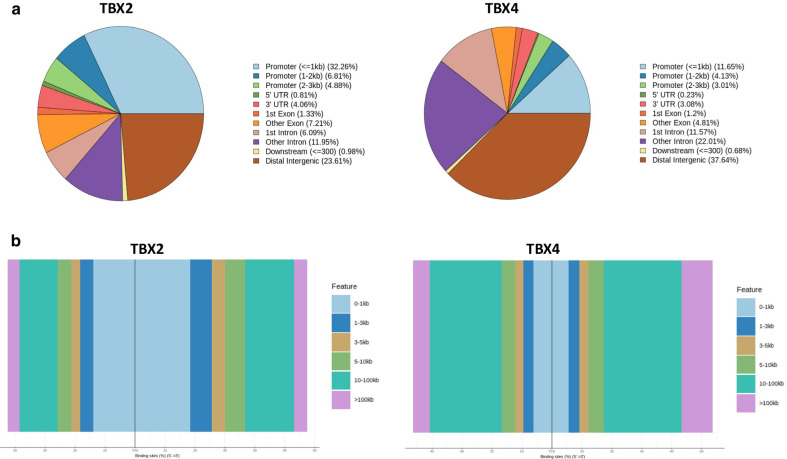
Genome-wide annotation of TBX2 and TBX4 binding sites in IMR-90 cells. **a** Pie charts showing the distribution of the TBX2 (left panel) and TBX4 (right panel) binding sites according to peak location across different genomic regions in the human genome. **b** Distribution of TBX2 (left panel) and TBX4 (right panel) binding sites relative to the nearest transcription start site (TSS) across the human genome

Genomic annotations of TF binding peaks identified for both TFs were analyzed and compared along the human genome to determine whether TBX2 and TBX4 interact with different regions. The genomic locations of enriched peaks, annotated to the most proximal transcription start site (TSS), showed a wide distribution pattern (Fig. 1). In total, 43.95% of TBX2 binding sites were located near gene promoters (including 32.26% sites mapped <  = 1 kb to the TSS) and 24.59% sites were located in intergenic regions, while 18.04%, 8.54%, and 4.87% map to introns, exons, and untranslated regions (UTRs), respectively (Fig. 1). Conversely, the majority of TBX4 binding sites were mapped to intergenic intervals (38.32%) followed by introns (33.58%), near-promoter regions (18.79%, including 11.65% sites mapped ≤ 1 kb to the nearest TSS), exons (6.01%), and UTRs (3.31%) (Fig. 1).

TBX2 and TBX4 binding peaks annotated to the nearest gene, classified according to the ENSEMBL biotypes, revealed that some genes have more than one TBX2 or TBX4 binding sites. Among the 14,322 enriched peaks detected for TBX2, 7346 different genes were identified; 85.04% of them are protein genes, 6.26% lncRNAs, 6.09% miRNAs, 2.02% pseudogenes, and 0.59% other RNA biotypes, including miscRNAs, scaRNAs, snoRNAs, snRNAs, and rRNAs (Additional file 2). Similarly, 1862 TBX4 binding sites were annotated to 1456 different genes, including 83.37% protein coding genes, 6.23% lncRNAs, 6.30% miRNAs, 3.44% pseudogenes, and 0.66% genes classified as other RNA biotypes (Additional file 2).

### Overrepresentation of novel and known TF binding sites within enriched peaks for TBX2 and TBX4

The analysis of the binding motifs for TBX2 revealed several overrepresented novel sequences (Table 1), including TWYTYKGKAKKYTKAGKCRRGASRRTGKCK, TGCAGTGGCGCGATCTCGGCTCACTGCAAG, and CTGGGMAGTGAGGRGCKYMKCWSCCSG found in 257, 156, and 469 of the TBX2 peaks, respectively. Amongst the relatively short motifs, the most significant were CNGGRA, CTCCWCAC, and AATGGCGT observed in 13,048, 901, and 193 TBX2 peaks. The detected sequences matched several known binding motifs, including, MA0806.1 (TBX4), 0144.2 (STAT3), MA1100.1 (ASCL1), MA0830.1 (TCF4), MA0690.1 (TBX21), and MA0807.1 (TBX5). The most enriched known motifs detected within the 100 nt TBX2 peak summits were MA0497.1 (MEF2C), MA0136.2 (ELF5), and MA1122.1 (TFDP1) (Additional file 3).

**Table 1:**
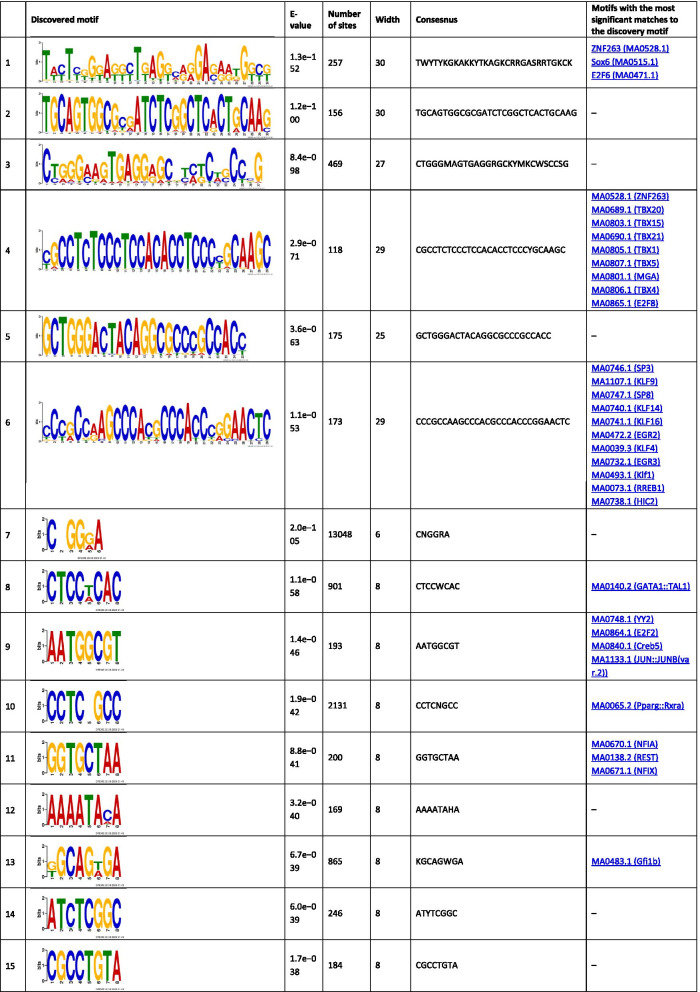

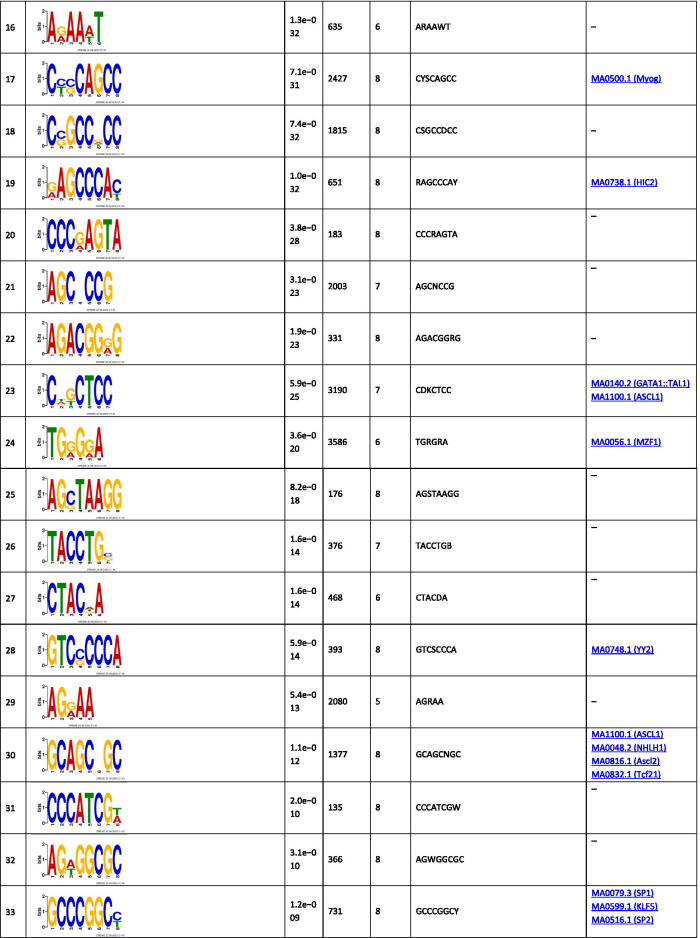

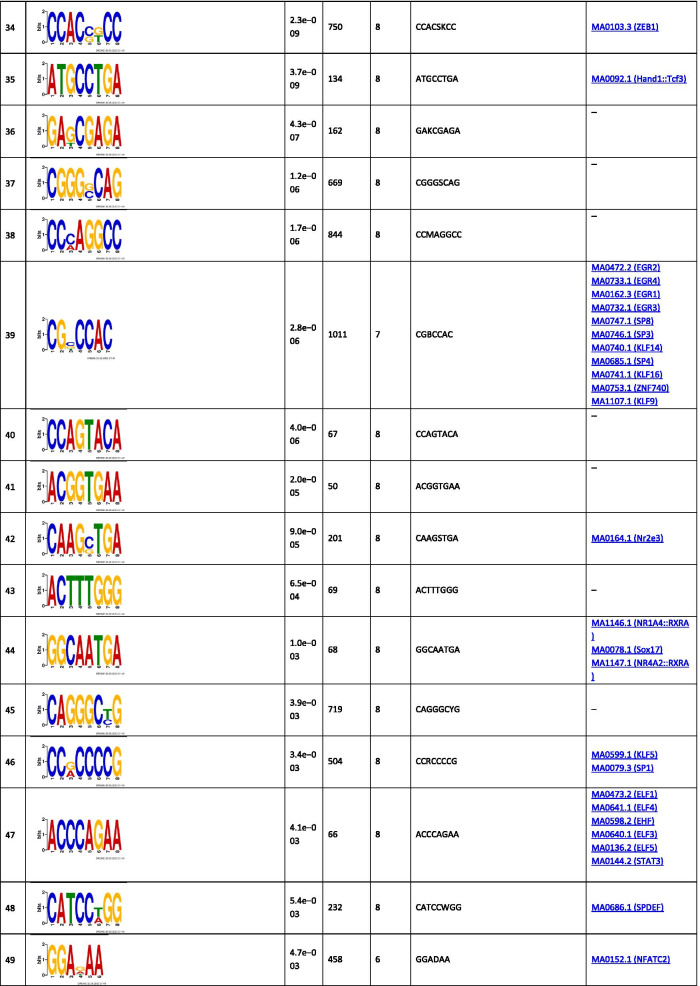

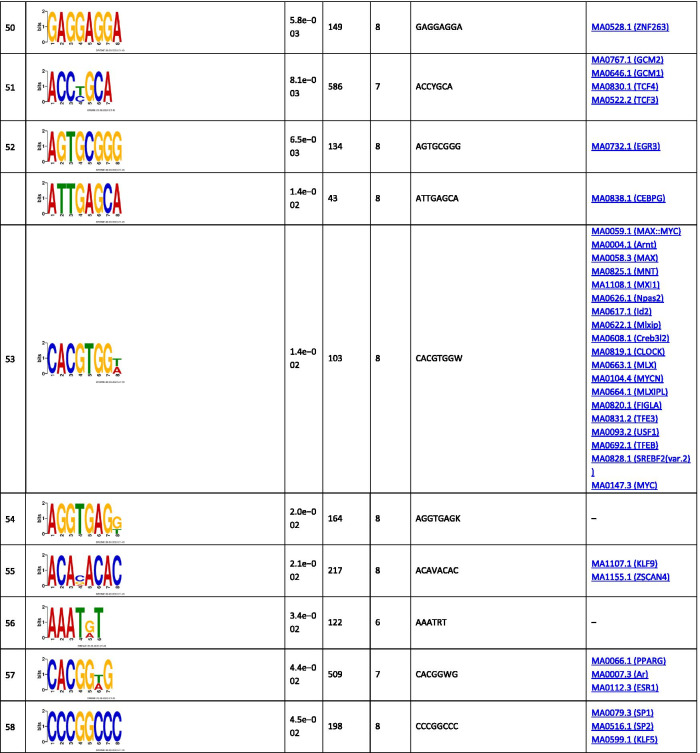
The significant motifs (E-value ≤ 0.05), found for the TBX2 peaks using the MEME and DREME programs, are clustered by similarity and ordered by E-value

Similar analyses performed for TBX4 revealed six long overrepresented novel motifs, including TCCMWYRGMTTGSRRKGARRTGTRGAGGRA, GCCGGGCAGAGRCGCTCCTCACYTCC, and GGGCAGTGAGGGGCTTAGCACCYGGGCCA as the most significant ones found in 61, 47, and 36 peaks, respectively (Table 2). Amongst the short motifs, the most significant were CNGGRA, GGTKTGGA, and GCCTCTSC overrepresented in 1753, 75, and 172 TBX4 peaks. A comparison of the identified novel motifs with the known TF binding sequences revealed that they matched the consensus binding of other TFs, including MA0690.1 (TBX21), MA0688.1 (TBX2), and MA0689.1 (TBX20).

**Table 2:**
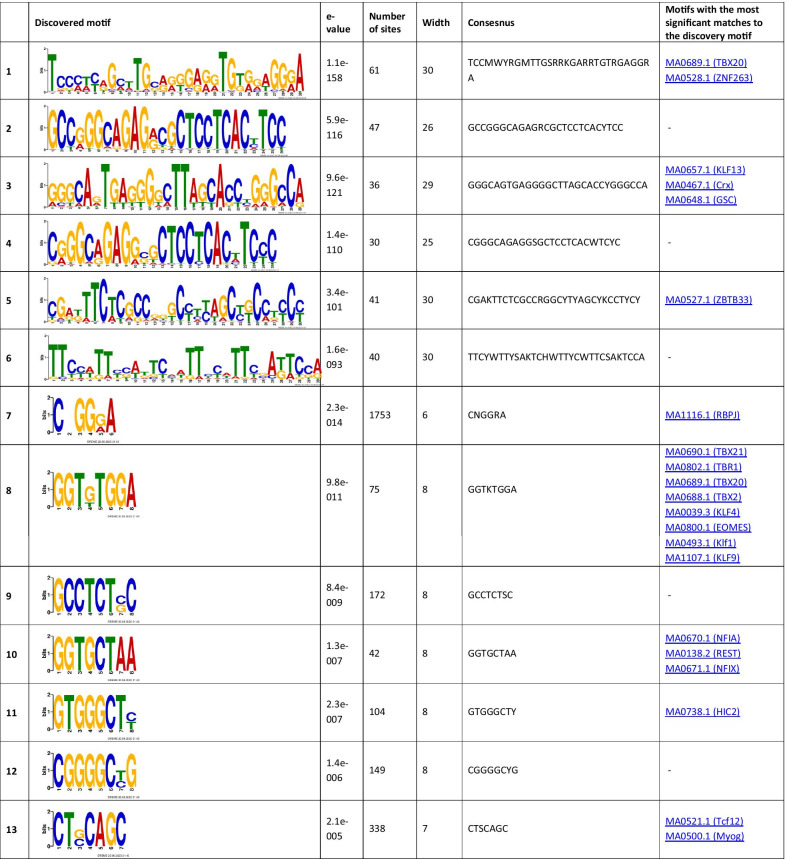

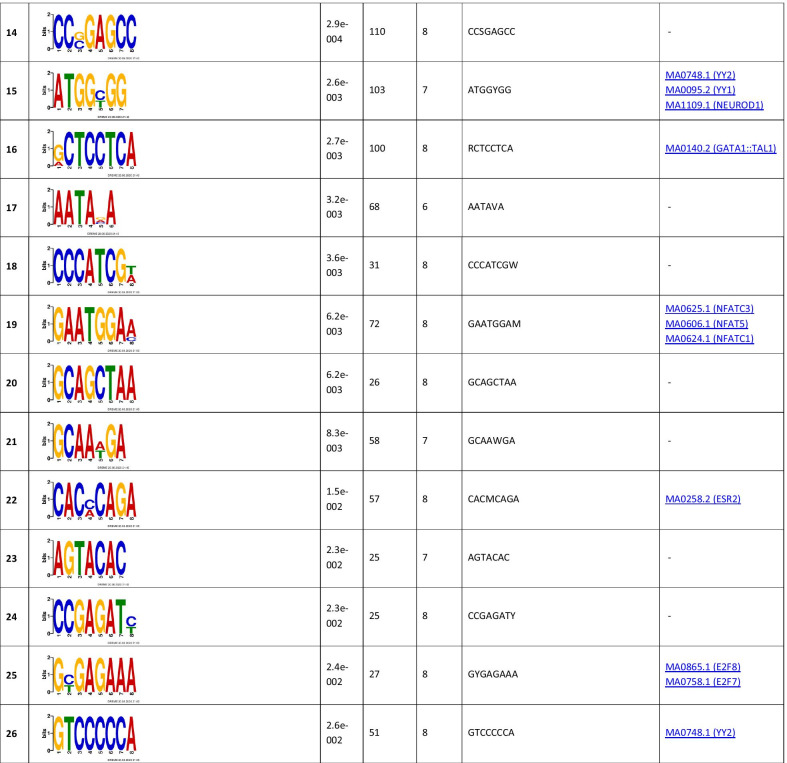
The significant motifs (E-value ≤ 0.05), found for theTBX4 peaks by the MEME and DREME programs, are clustered by similarity and ordered by E-value

The enrichment results for the known motifs within TBX4 binding sites were the strongest for MA1116.1 (RBPJ), MA1122.1 (TFDP1), and MA0138.2 (REST) (Additional file 3).

### Transcriptional regulation of genes involved in lung development

**Fig. 2:**
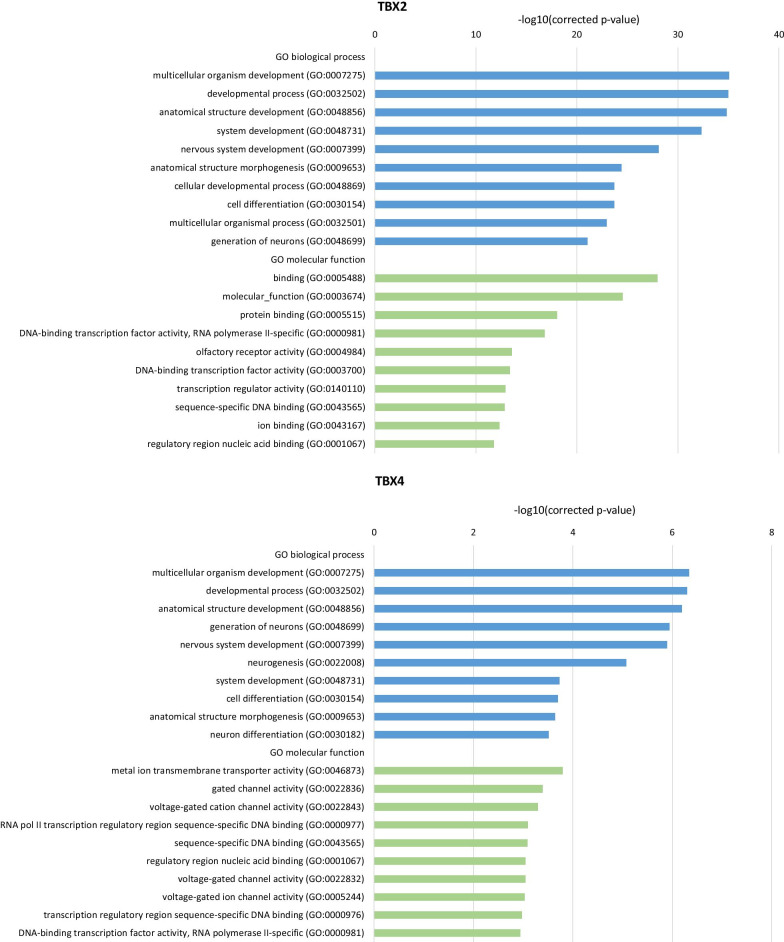
Gene ontology analysis of the genes located in close proximity to TBX2 and TBX4 binding sites. A bar chart of the most significant gene ontology (GO) biological processes (blue) and molecular function (green) terms for genes that are located around enriched TBX2 (upper panel) and TBX4 (bottom panel) binding

To identify the functional annotations of genes located in the close proximity to the ChIP-seq peaks, 7346 and 1456 genes for TBX2 and TBX4, respectively, were subjected to the GO enrichment analyses. We found 675 enriched GO terms for TBX2 and 96 for TBX4 (Additional file 4). For both TF gene sets, the most overrepresented GO biological processes included multicellular organism and anatomical structure development (Fig. 2). Within GO molecular function, the most enriched ones were associated with protein or nucleic acid binding and transcription regulatory activity for TBX2 (Fig. 2), and channel activities and nucleic acid binding for TBX4 (Fig. 2).

Overrepresentation analyses of the molecular pathways among the enriched ChIP-seq peaks were carried out to understand the high-level functions of the identified genes. Numerous significantly overrepresented pathways were detected, several of which are related to lung development (Additional file 5). Prominent pathways associated with TBX2 in IMR-90 cells include Wnt signaling, axon guidance, VEGFA-VEGFR2 signaling, Notch signaling, and hedgehog signaling. The Wnt signaling, axon guidance, and differentiation pathways were enriched for TBX4.

**Fig. 3:**
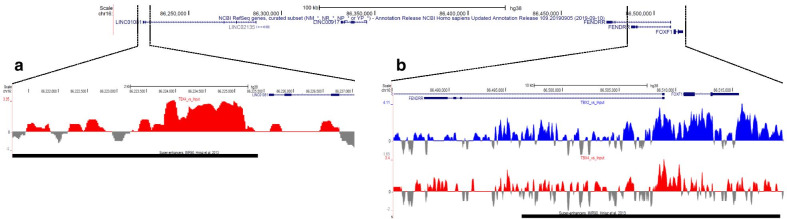
Schematic representation of *FOXF1* and* FENDRR* locus on chromosome 16q24.1. The 16q24.1 region depicting **a** TBX4 (red) binding sites within *FOXF1* and *FENDRR* lung-specific core enhancer interval and **b** TBX2 (blue) and TBX4 (red) binding sites within the *FOXF1* and *FENDRR* locus detected using ChIP-seq in IMR-90 cells. Input signals are shown in grey. Binding sites for both transcription factors partially overlap super-enhancer region (black bar) previously identified in IMR-90 cells [31]

**Fig. 4:**
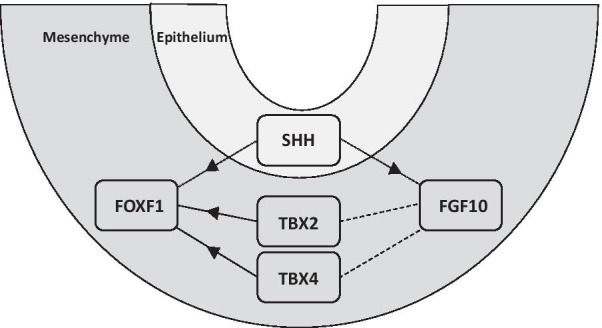
Schematic depiction of TBX4-FGF10 and SHH-FOXF1 signaling regulating branching morphogenesis. Dark grey denotes epithelium and light grey represents mesenchyme. Suggested regulation of FOXF1 by TBX4 and TBX2 (solid lines) is based on TF binding pattern detected in ChIP-seq experiment presented in this work. Interactions between epithelial SHH and mesenchymal FOXF1 and FGF10 (dotted lines) as well as T-Box TF and FGF10 (dashed lines) are proposed based on the literature data [5, 16, 47]. Arrowheads indicate direction of protein–protein interactions

Of note, the significant enrichment of the TF binding sites was identified in regions located within or near the components of the known TBX4-FGF10 signaling, including *TBX2*, *TBX4*, *TBX5*, *BMP4* (TBX2), *WNT2*, and *SPRY2* (TBX4). In addition, TBX4 binding sites were found in the *FOXF1* and *FENDRR* lung-specific core enhancer interval (Fig. 3a**)**. Both TBX2 and TBX4 binding sites were enriched within *FENDRR*, and TBX2 binding sites within the *FOXF1* intron and around 3′UTR of *FOXF1* (Fig. 3b). Schematic depiction of TBX4-FGF10 and SHH-FOXF1 signaling regulating branching morphogenesis is presented in Fig. 4.

Intersection of the detected peaks with known super-enhancer intervals previously identified in IMR-90 cells showed 544 and 19 overlapped regions for TBX2 and TBX4.

## Discussion

The molecular pathways that control each of the five histologically-defined stages of lung organogenesis have been extensively analyzed [3, 32]. However, the unique crosstalk between TFs and genes implicated in lung formation is still poorly understood.

The ChIP-seq analyses revealed 14,322 and 1862 potential targets for TBX2 and TBX4 across the human genome in IMR-90 cells, including 991 binding sites shared by both of them. The annotation of the TBX2 and TBX4 peaks to the most proximal TSS showed a distinct biding pattern. The association of TBX2 with chromatin may have a most important role at gene promoters and distal intergenic regions. In contrast, TBX4 interactions are most prominent within intergenic regions away from genes and intronic intervals. The results of annotation of enriched peaks to the nearest gene classified based on gene biotype suggest that both TFs may control not only protein coding genes but also non-coding RNAs, including lncRNAs. These findings are important, particularly due to the relevance of non-coding RNAs in lung development [33].

The functional classification of genes overlapping TBX2- and TBX4-binding peaks indicated that significant biological processes and molecular functions, including multicellular organism development, developmental process, and anatomical structure development as well as nucleic acid binding, can be regulated by TBX2 and TBX4. Interestingly, further analyses of the genes overlapping the identified peak intervals revealed enrichment for a number of pathways that interact with each other and orchestrate lung development.

The Wnt signaling, enriched for both TFs, has a diverse role in regulating cell functions during morphogenesis. The Wnt/β-catenin pathway orchestrates cell fate decisions and differentiation of lung cells and is thus required for lung development in utero [34]. In the postnatal period, the Wnt signaling pathway controls both lung tissue homeostasis and repair, and its disruption leads to asthmatic remodeling or tissue fibrosis [35, 36]. Several animal studies revealed that modulation of the Wnt pathway by gene knockouts resulted in early lethality often associated with impaired respiratory development due to prevention of distal lung buds formation [37]. Recently, we also reported variants in *CTNNB1* and *TBX4*, encoding crucial members of the Wnt and FGF signaling, in a newborn with abnormal lung growth, PAH, severe microcephaly, and spasticity, suggesting that mutations in these genes could act synergistically resulting in a lethal respiratory failure during the neonatal period [38].

Another identified pathway associated with lung bud formation and enriched in TBX2 and TBX4 binding sites in our analyses is axon guidance. Whereas genes involved in this pathway were initially described in nervous system as regulators of neural network formation, many recent studies indicated an essential role of axon guidance in regulating extracellular matrix interactions during morphogenesis [39]. Axon guidance proteins, including semaphorins or ephrins, act at early steps of lung branching when they shape the architecture of the lung bud and are also responsible for normal alveolar development [39].

Among the prominent pathways potentially regulated by TBX2 in studied IMR-90 cells are VEGF and Notch signaling, which control pulmonary vasculogenesis in fetal lungs [40, 41]. Interestingly, VEGF inhibition and disruption of Notch signaling in mice were shown to impair alveolarization [42, 43]. Deregulation of VEGF or Notch signaling can result in PAH [44] and other respiratory diseases, including pulmonary fibrosis [45, 46].

In this study, one of the most significantly enriched molecular pathways for TBX2 is SHH signaling. In mice, SHH progressively limits lung bud outgrowth by downregulation of *Fgf10* expression in the distal mesoderm [3]. On the other hand, SHH-induced *Hhip1* inhibits SHH signaling and allows for local *Fgf10* expression and bud development in lung branching zones [3]. Through regulation of GLI processing in lung epithelium, SHH also regulates expression of the *Foxf1* and *Tbx* genes, promoting lung proliferation [47]. Mice studies showed that TBX4 may indirectly control *Fgf10* expression [16]. However, we found no evidence of TBX4 binding in the vicinity of *FGF10*, suggesting that FGF10 may be regulating *TBX4*.

Interestingly, our ChIP-seq analyses revealed that TBX4 specifically binds to the recently narrowed down *FOXF1* lung-specific distal core enhancer interval [6, 48] and that TBX2 binds to the *FOXF1* intron, also featuring enhancer activity, and *FOXF1* 3′UTR*,* suggesting that it may also regulate *FOXF1* expression. In addition, TBX2 and TBX4 binding sites were found to be enriched within *FENDRR*. These data indicate that the TBX4-FGF10 cascade may interact with the SHH-FOXF1 signaling in human lung development. The crosstalk between these pathways may also partially explain some similarities in histopathological appearance of TBX4- or FOXF1-derived LLDDs at lung biopsy or autopsy, demonstrating a spectrum of developmental arrest in lung growth and/or maturation.

The analyses of DNA sequences within the binding sites for TBX2 or TBX4 showed overrepresentation of several novel and known motifs. Among the novel sequences were those that also matched binding motifs of the known TFs, including TBX21, STAT3, ASCL1, and TCF4. While TBX21 is not known to be involved in lung morphogenesis, mice studies showed that *Tbx21* deficiency induces a phenotype reminiscent of human asthma [49]. STAT3 is associated with maintenance of surfactant homeostasis and lung function during hyperoxia [50] and ASCL1 is critical for the development of pulmonary neuroendocrine cells [51]. TCF4 is a part of canonical Wnt/catenin-β signaling and it forms a complex with LEF and catenin-β in the nucleus, activating Wnt target genes required for lung development [52].

Analyses of the known TF motifs within the TBX2 and TBX4 binding peaks in IMR-90 cells revealed a strong enrichment for TFDP1. This TF binds DNA of the E2F family members that regulate cell cycle [53]. Loss of *Tfdp1* in mice induced pulmonary artery remodeling in response to hypoxia [54]. The Rbpj TF, enriched in TBX4 binding sites, is the effector of Notch signaling regulating the balance between ciliated and secretory cell fates during airway differentiation [43], while ELF5, enriched in TBX2 binding sites, is an FGF-sensitive TF that can regulate differentiation of epithelial cells in the developing lung [55].

The identification of the known TFs motifs involved in lung function within the detected TBX2 and TBX4 binding sequences indicates that they both can interact with other TFs and thus cooperatively mediate lung development. In addition, the genomic loci marked previously as super-enhancers in IMR-90 cells [31] were enriched with the TBX2 (544) and TBX4 (19) marks, suggesting that both TFs play a role in cell-specific transcriptional regulation of genes involved in lung development also via super-enhancers.

## Conclusions

In summary, our results imply that TBX2 and TBX4 together or separately regulate genes mediating branching morphogenesis and vascular development in human lungs. ChIP-seq analyses enabled us to map physical interactions between DNA and TBX2 or TBX4 in IMR-90 human fetal lung fibroblasts, facilitating a better understanding of the specific regulatory networks maintaining tissue homeostasis required for proper lung development. By identifying enrichment of TBX2 and TBX4 DNA target sites within or around genes from molecular pathways as well as known TFs involved in lung development, we highlight the complexity of transcriptional regulation driven by both studied TFs. We also show that the TBX4-FGF10 cascade might interact with the SHH-FOXF1 signaling orchestrating lung development, providing further indications that disruption of this crosstalk during morphogenesis can play a role in the etiology of lethal lung developmental disorders. The detailed molecular mechanisms of its regulation require additional investigation. To gain further functional insights into TBX4-FGF10 signaling, we plan to use RNA sequencing in lung autopsy/biopsy tissues from patients with various LLDDs due to variants in TBX4 or FGF10.

## Supplementary Information

**Additional file 1:** Enriched binding sites for TBX2 and TBX4 determined by ChIP-seq analysis in IMR-90 cells.

**Additional file 2:** Gene biotype distribution of genes with at least one TBX2 or TBX4 binding site in their surrounding areas. Pie charts showing the distribution of the annotated genes for TBX2 (left panel) and TBX4 (right panel) binding sites, according to their ENCODE biotype.

**Additional file 3:** The list of known motifs enriched within TBX2 and TBX4 100 nt peak summits. Analysis was performed using AME tool. Only motifs with enrichment E-values no greater than 10 was reported (the E-value is the motif p-value multiplied by the number motifs in the input).

**Additional file 4:** Gene ontology (GO) analysis of the neighboring genes associated with the ChIP-seq peaks for TBX2 and TBX4. Analysis was performed for GO biological process and GO molecular function terms using PANTHER tool. The GO terms with a corrected p-value < 0.05 (FDR) were considered significantly enriched.

**Additional file 5:** Overrepresented molecular pathways associated with genes located near TBX2 and TBX4 binding sites. Analyses were performed using ConsensusPathDB tool. Only pathways with p-value ≤ 0.05 and sharing at least two genes with the ChIP-seq gene sets were analyzed.

## Data Availability

The datasets used and/or analyzed during the current study are available from the corresponding author on reasonable request.

## Abbreviations

AcDys: Acinar dysplasia
ACDMPV: Alveolar capillary dysplasia with misalignment of pulmonary veins
CAD: Congenital alveolar dysplasia
ChIP-Seq: Chromatin immunoprecipitation followed by next-generation sequencing
CNV: Copy number variant
FGF10: Fibroblast growth factor 10
FOXF1: Forkhead box protein F1
LLDD: Lethal lung developmental disorder
PAH: Pulmonary arterial hypertension
SNV: Single nucleotide variant
SHH: Sonic hedgehog
TBX2/4/5: T-box transcription factor 2/4/5
TF: Transcription factor
UTR: Untranslated region
