# Supplementary material for: Potential interactions between the TBX4-FGF10 and SHH-FOXF1 signaling during human lung development revealed using ChIP-seq
Source: Respir Res. 2021 Jan 21;22:26. doi: 10.1186/s12931-021-01617-y (PMC7818749; doi:10.1186/s12931-021-01617-y)
Supplement: Supplementary file 2 — Additional file 2: Gene biotype distribution of genes with at least one TBX2 or TBX4 binding site in their surrounding areas. Pie charts showing the distribution of the annotated genes for TBX2 (left panel) and TBX4 (right panel) binding sites, according to their ENCODE biotype. [file 12931_2021_1617_MOESM2_ESM.docx]

**Additional file 2.**


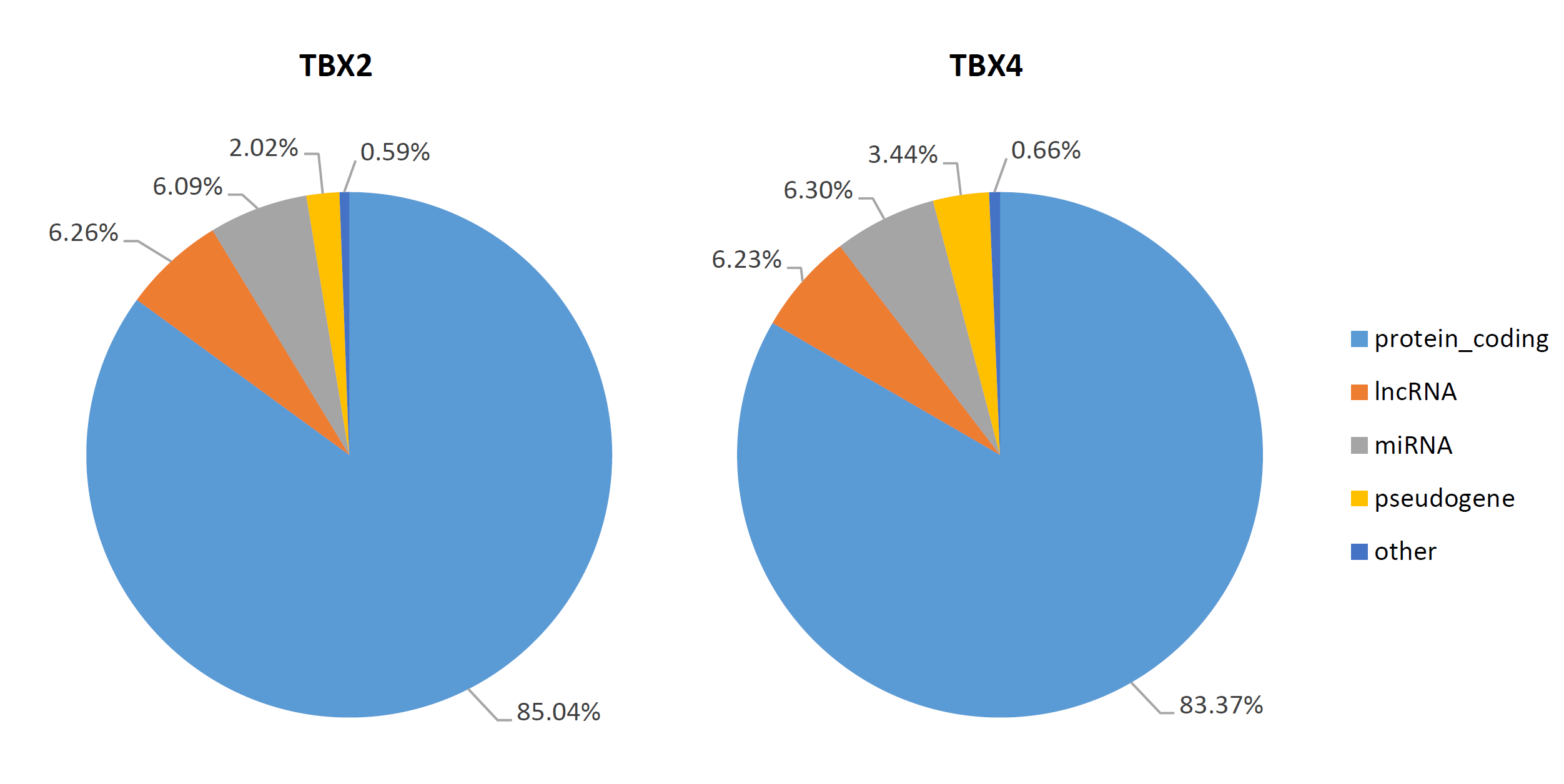


**Gene biotype distribution of genes with at least one TBX2 or TBX4 binding site in their surrounding areas.** Pie charts showing the distribution of the annotated genes for TBX2 (left panel) and TBX4 (right panel) binding sites, according to their ENCODE biotype.
